# An effective UFLC–MS/MS method used to study pharmacokinetics of major constituents of Fukeqianjin formula in rat plasma

**DOI:** 10.1186/s13020-020-00347-5

**Published:** 2020-07-25

**Authors:** Kanghua Wang, Lu Liu, Yanfang Yang, Xiaoyan Liu, Lei Zhang, Wei Xu, Yingtao Zhang, Xiuwei Yang, Peng Zhang, Kaifeng Peng, Yun Gong, Nifu Liu

**Affiliations:** 1 State Key Laboratory of Natural and Biomimetic Drugs, Department of Natural Medicines, School of Pharmaceutical Sciences, Peking University Health Science Center, Peking University, No. 38, Xueyuan Road, Haidian District, Beijing, 100191 China; 2 Zhuzhou Qianjin Pharmaceutical Co., Ltd, Zhuzhou, 412000 China

**Keywords:** Fukeqianjin formula, Pharmacokinetics, UFLC–MS/MS

## Abstract

**Background:**

Fukeqianjin formula (FKQJF) is a Chinese medicine prescription, which has been widely used individually or in combination with other western medicine for the treatment of various gynecological inflammatory diseases, including chronic cervicitis, chronic pelvic inflammatory disease and endometritis, so on and so force.

**Methods:**

The ultra-fast liquid chromatography coupled with triple quadrupole tandem mass spectrometry (UFLC–MS/MS), a quick and efficient method was established and applied to quantify the major constituents of Fukeqianjin formula in rat plasma, and its pharmacokinetics of oral absorption was studied. Nineteen components in Fukeqianjin formula were detected and identified as the major compounds absorbed into the blood according to their chromatographic behavior, molecular weight, ion fragments and other information of these compounds. Furthermore, the plasma drug concentration–time curves were established and the related kinetic parameters were analyzed.

**Results:**

The results showed that all the 19 compounds could be rapidly absorbed by the gastrointestinal tract, the plasma drug concentration of most compounds could reach a peak at around 1–2 h, and the double-peaks on behalf of the enterohepatic circulation were found in most drug concentration–time curves. The method used in this experiment was validated comprehensively including specificity, linearity, precision, accuracy, stability, matrix effect, and recovery.

**Conclusions:**

These results showed that the developed method was suitable for pharmacokinetic analysis of the main components of Fukeqianjin formula in rat plasma, and may provide useful information for the subsequent distribution studies in vivo.

## Background

Traditional Chinese medicine (TCM) prescriptions are usually made up of several medicinal herbals according to certain mass ratios guided by traditional Chinese medicine theory, not simply the addition of herbs. It is well known that the multiple constituents contribute to the therapeutic and synergistic effects of traditional Chinese medicine (TCM), and the chemical components absorbed into blood have more possibilities of showing pharmacological activity. Furthermore, the efficacy of TCM does not simply equal the sum of the efficacies of all active components. Therefore, identification of the absorbed chemical components and metabolites are therefore very important for elucidating the therapeutic material basis and action mechanism of TCM.

Fukeqianjin formula (FKQJF), a Chinese medicine prescription, consists of eight Chinese medicinal materials including *Moghaniae* Radix*, Rosae Laevigatae* Radix*, Andrographis Herba, Mahoniae Caulis, Zanthoxyli* Radix et Caulis*, Angelicae Sinensis* Radix*, Spatholobi Caulis*, and *Codonopsis* Radix [1]. FKQJF has been widely used individually or in combination with other western medicine for the treatment of various gynecological inflammatory diseases, including chronic cervicitis, chronic pelvic inflammatory disease, endometritis, etc. [2–4]. Modern pharmacological studies and clinical practices have demonstrated its anti-inflammatory, bacteriostasis, and analgesic effects [5, 6]. Our previous study on the chemical components of FKQJF showed that it contained numerous diverse compounds, including flavonoids, alkaloids, diterpenes, phenols, phthalides, and phenylpropanoids, etc. [7]. The diterpenes (neoandrographolide, andrographolide, 14-deoxy-11,12-didehydroandrographolide and andrograpanin) and flavonoids (7-*O*-methylwogonin and skullcapflavone I) are considered to be the main bioactive components of *Andrographis Herba*; the alkaloids (methyl-5-hydroxy-2-pyridinecarboxylate, acortatarin A, 9-epi-acortatarin A, jatrorrhizine, palmatine, and berberine) and phenol (3,4,5-trimethoxyphenyl-1-*O*-β-d-glucopyranoside) originated from *Mahoniae Caulis*, especially jatrorrhizine, palmatine, and berberine are considered to be the primary bioactive compounds of *Mahoniae Caulis.*; the phthalide (Z-ligustilide) and phenylpropanoid (ferulic acid) are the main effective ingredients isolated from *Angelicae Sinensis* Radix; the flavonoid (genistin) is the effective ingredient isolated from *Moghaniae* Radix and the flavonoids (ononin and naringenin) originated from *Spatholobi Caulis*. These complex ingredients are the main bioactive components of FKQJF and show a variety of pharmacological effects. For example, Liu et al. tested the anti-inflammatory activities of neoandrographolide and andrograpanin, which could significant suppressed the level of NO and TNF-α in LPS-induced inflammatory model [8, 9]. As for the alkaloids, jatrorrhizine, palmatine, and berberine were reported exhibited various activities, such as anti-inflammatory, antinociceptive and anti-pyretic activities [10, 11], and being used to help treat several diseases in clinic. Ononin, naringenin and genistin are the mainly flavonoids isolated from FKQJF, which exhibited significant anticancer, anti-inflammatory, and antibacterial activities, especially for the estrogen-like activity and being used for the alleviation of menopause symptoms [12, 13]. However, there is still no report focused on the in vivo absorbed components of FKQJF. Accordingly, it is necessary to establish a rapid and sensitive analytical method for detection and quantification of the absorbed components of FKQJF.

Ultra-fast liquid chromatographic coupled with electrospray ionization triple quadrupole tandem mass spectrometry (UFLC–MS/MS) technology is suitable for the analysis of the complex system of TCM, especially for the low concentration components. The main purpose of this study is to establish and validate a rapid, accurate, precise, sensitive, and selective UFLC–MS/MS for the identification and quantification of 19 major constituents of FKQJF in rat plasma after oral administration of FKQJF. It was expected that the results of this study could provide helpful information for further understanding the relationship between the chemical constituents and pharmacological activity of FKQJF.

## Materials and methods

### Materials and reagents

*Moghaniae* Radix is the dried roots of *Moghania macrophylla* (Willd.) O. Kuntze and was obtained from Xishuangbanna Dai Autonomous Prefecture of Yunnan province of China. *Rosae Laevigatae* Radix is the dried roots of *Rosa laevigata* Michx. and was obtained from Yongshun county of Hunan province of China. *Andrographis Herba* is the dried aerial parts of *Andrographis paniculata* (Burm. f.) Nees and was gathered from Suixi county of Zhanjiang city in Guangdong province of China. *Mahoniae Caulis* is the dried stems of *Mahonia bealei* (Fort.) Carr. and was purchased from Yunnan province of China. *Zanthoxyli Caulis* is the dried stems of *Zanthoxylum dissitum* Hemsl. and was purchased from Yanling county of Zhuzhou city in Hunan province of China. *Angelicae Sinensis* Radix is the dried roots of *Angelica sinensis* (Oliv.) *Diels* and *Codonopsis* Radix is the dried roots of *Codonopsis pilosula* (Franch.) Nannf., both were purchased from Longxi county of Gansu province of China. And *Spatholobi Caulis* is the dried vine stem of *Spatholobus suberectus* Dunn and was harvested from Pu’er city, Yunnan province of China. All the crude drugs were identified by Prof. Xiu-Wei Yang at the School of Pharmaceutical Sciences, Peking University Health Science Center, Peking University. Voucher specimens were deposited at the herbarium of Zhuzhou Qianjin Pharmaceutical Co., Ltd. (Hunan, China) and school of Pharmaceutical Sciences, Peking University (Beijing, China).

**Fig. 1:**
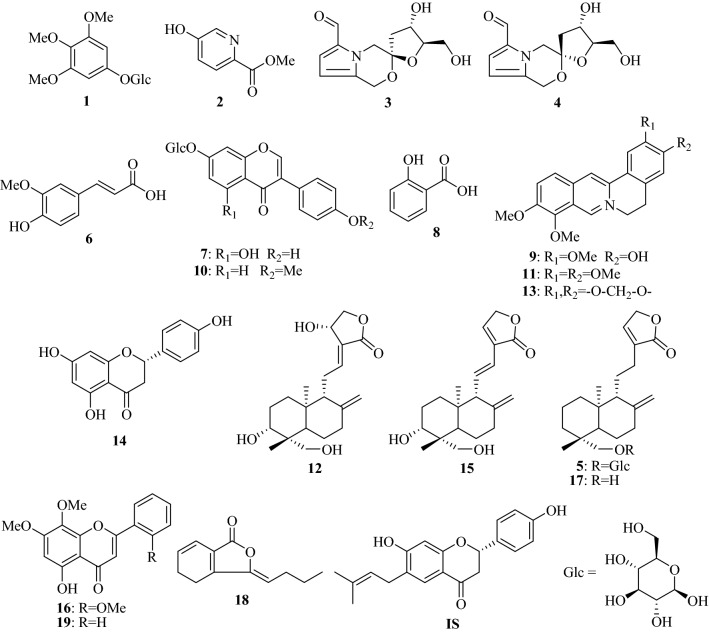
Chemical structures of analytes **1**–**19** and internal standard (I.S.)

Reference compounds including 3,4,5-trimethoxyphenol-1-*O*-β-d-glucopyranoside (**1**), 5-hydroxypicolinic acid methyl ester (**2**), acortatarin A (**3**), 9-epi-acortatarin A (**4**) were isolated from *Mahoniae Caulis* [14], neoandrographolide (**5**), *trans*-ferulic acid (**6**), genistin (**7**), salicylic acid (**8**), jatrorrhizine (**9**), palmatine (**11**), andrographolide (**12**), berberine (**13**), 14-deoxy-11,12-didehydro-andrographolide (**15**), panicolin (**16**), andrograpanin (**17**), Z-ligustilide (**18**), and 7-*O*-methylwogonin (**19**) were isolated from the extracts of Fukeqianjin Formula as described in our previous report [7], ononin (**10**) and naringenin (**14**) were isolated from *Spatholobi Caulis* [15]. The internal standard (I.S.) bavachin was isolated from the mature fruits of *Psoralea corylifolia* L. [16]. Their purity was determined to be above 98% by LC–MS and the chemical structures are shown in Fig. 1.

Heparin sodium injection (Lot# 20150602) was purchased from Tianjin Biochemical Pharmaceutical Co., Ltd. (Tianjin, China). Methanol (MeOH), acetonitrile (ACN) and formic acid of LC–MS grade were provided by Fisher Scientific Inc. (Pittsburgh, PA, USA). Deionized water (18.2 MΩ cm) was purified by Millipore Alpha-Q water purification system (Millipore, Bedford, MA, USA).

### Animals

Rat experiment was carried out based on following the guideline of the Care and Use of Laboratory Animals in Beijing that was approved by Animal Care and Use Committee of Peking University (Approval No. LA 2018143, 1 March 2018). Male Sprague-Dawley (SD) rats with the weight of 220 ± 20 g, were provided by Laboratory animal center of Peking University Health Science Center. These rats were raised in a standard environment with temperature of 24 ± 2 °C, relative humidity of 60 ± 5%, and LD 12:12 cycles. They could acclimatize the environment for a week before the experiment, free drinking water and conventional feed were normally supplied during this period.

### Preparation of FKQJF extract

FKQJF extract was prepared and freeze-dried by using standard method as described previously [1] and obtained from Zhuzhou Qianjin pharmaceutical Co., Ltd. Chiefly, 21 g dried FKQJF extract was added into moderate amount of distilled water to dissolved completely by ultrasonic treatment, then diluted to get a suspension of 0.42 g/mL.

### Pretreatment of plasma samples

Aliquots of 100 μL plasma samples were mixed with 10 μL I.S. samples (1 μg/mL, in MeOH), then added with 500 μL ACN, respectively, and vortexed for 5 min, centrifugated at 10,000 rpm for 10 min, the supernatants were transferred into clean vials and dried in a vacuum centrifugal concentrator at 1200 rpm, 37 °C. After that, 100 μL ACN was added and ultrasonic (frequency 40 kHz, power 250 W) extracted for 5 min to dissolve the dry residue, vortexed for 1 min and centrifugated at 10,000 rpm for 5 min, 1 μL of each final supernatant was injected into UFLC–MS/MS for analysis.

### Instrument and analytical conditions

An 8050 UFLC–MS/MS system equipped with LC-30A binary pump, a SIL-30AC autosampler, a SPD-M30A PDA detector, a CTO-20AC column oven, and a 8050 triple quadrupole mass spectrometer with an electrospray ionization source (ESI) was applied to detect and quantify FKQJF compounds in plasma samples. Data were acquired and processed with the software LabSolution (Ver. 5.6).

A Kinetex XB-C_18_ HPLC column (100 × 2.1 mm, 2.6 μm) linked with an equivalent guard column was used for chromatographic separation. The aqueous phase contained 0.2% formic acid was used as mobile phase A, and organic phase ACN as mobile phase B. The gradient elution method using in this program was: 3–5 min, 28–31% B; 5–7 min, 31–34% B; 7–9 min, 34–65% B; 9–10 min, 65–95% B; 10–11.5 min, 95% B. The flow rate of mobile phase, column temperature and autosampler temperature was 0.4 mL/min, 30 °C and 4 °C, respectively. The injection volume of each sample was set as 1 μL. The main MS spectrum parameters were set as follows: the flow rate of drying gas (N_2_) was 10.0 L/min; the atomized gas was 3.0 L/min and the heating gas flow rate was 10.0 L/min. The main temperatures of interface, desolvation and heat block was 300 °C, 250 °C and 400 °C, respectively. And, the interface and detector voltage were 3 kV and 1.8 kV. The specific parameter values corresponding to each compound were shown in Table 1.

**Table 1:** The information of compound ionization

Analyte	Retention time (min)	MRM transition (*m/z*)		Dwell time (msec)	Q_1_ pre bias (V)	Collision energy (V)	Q_3_ pre bias (V)
		Precursor ion	Product ion				
**1**	2.022	347.05	185.10	22	− 21	− 13	− 18
**2**	2.079	154.20	94.00	22	− 25	− 10	− 26
**3**	2.184	254.15	206.05	22	− 15	− 10	− 25
**4**	2.464	254.20	206.05	22	− 15	− 10	− 21
**5**	2.539	481.2	23.05	22	− 26	− 45	− 29
**6**	3.206	195.05	89.00	22	− 25	− 33	− 16
**7**	3.571	431.10	268.05	22	16	30	28
**8**	3.835	137.10	93.05	22	10	16	29
**9**	3.837	338.20	323.10	22	− 25	− 24	− 15
**10**	3.979	431.10	269.10	22	− 24	− 22	− 28
**11**	4.324	352.20	336.10	22	− 19	− 31	− 16
**12**	4.355	395.25	331.10	22	28	12	22
**13**	4.461	336.15	320.15	22	− 24	− 31	− 15
**14**	5.701	273.10	153.00	22	− 30	− 22	− 15
**15**	7.354	333.2	292.05	31	− 19	− 10	− 30
**16**	8.928	315.10	300.05	46	− 26	− 22	− 20
**17**	9.391	319.25	301.15	46	− 30	− 13	− 20
**18**	9.670	191.10	77.05	46	− 13	− 41	− 29
**19**	9.715	299.10	283.00	46	− 30	− 32	− 19
**I.S.**	9.186	325.15	149.00	46	− 30	− 35	− 20

Dwell time: residence time during an acquisition point; Q1 pre bias: voltage promotes the ionization of the precursor ion; Q3 pre bias: voltage promotes the ionization of the product ion

### Method validation

The analysis method established in this experiment was systematically examined for specificity, linear, precision, accuracy, stability, recovery and matrix effect in accordance with guidelines for the validation of quantitative analytical methods for biological samples in Guidance for Industry: Bioanalytical Method Validation [17].

#### Method specificity

The specificity of UFLC–MS/MS method was verified by analyzing and comparing the chromatograms generated by the following three groups: Six pure blank plasma samples added with 100 μL ACN; Six blank plasma samples added with 100 μL ACN containing mixed reference compounds and I.S.; Six plasma samples collected from FKQJF-administrated rats and prepared as described in the section of “Pretreatment of plasma samples”.

#### Linearity, and lower limits of detection and quantification

The standard curve of each compound was established by weighted least squares method (WLS) drawing the peak area ratios of compound/I.S. (*y*) versus the corresponding concentrations of each compound (*x*). Each of these compounds was diluted into a series of concentrations in MeOH, followed by twofold dilution enable to cover all the range of plasma drug concentrations. The initial concentration of each compound in linear range was: 625 ng/mL for **1**, **2**, **3**, **4**, **5**, **8**, **10** and **14**; 743.75 ng/mL for **6**; 656.25 ng/mL for **7**; 200 ng/mL for **9**; 303.13 ng/mL for **11**; 768.75 ng/mL for **12**; 350 ng/mL for **13**; 312.5 ng/mL for **15**; 637.5 ng/mL for **16**; 768.75 ng/mL for **17**; 518.75 ng/mL for **18**; 450 ng/mL for **19**. The lower limit of detection (LLOD) and lower limit of quantification (LLOQ) were calculated according to the signal-to-noise ratio (*S*/*N*) of 3 and 10, respectively.

#### Precision and accuracy

The precision of intra-day and inter-day was obtained by calculating low, medium and high plasma concentrations of reference compounds in blank plasma. First, the low, medium and high concentrations of reference compounds was added in each blank plasma sample, six samples were prepared in parallel at each concentration and processed according to the section of “Pretreatment of plasma samples”. The intra-day and inter-day precision were investigated by repeating this experiment six times in 1 day, and repeating once for 3 days, respectively. The accuracy was evaluated by calculating the consistency of the measured concentration with the initial concentration.

#### Stability

Six replicates of low, medium and high concentrations of quality control (QC) samples were used to verify the stabilities under different conditions including: place at room temperature for 8 h, freeze (− 20 °C) and thaw for three times, and store at − 20 °C for 20 days.

#### Recovery and matrix effect

For recovery test, the blank plasma sample (100 μL) was mixed with 100 μL ACN containing low, medium or high concentration of the reference compounds and I.S., extracted with 500 μL ACN and further processed as described in the section of “Pretreatment of plasma samples”, and the peak area of each compound was labeled as A. For recovery control and matrix effect test, the blank plasma sample was replaced with 100 μL ACN containing the same three concentrations of the reference compounds and the peak area of each analyte was signed as B. For matrix effect control, the blank plasma sample was first extracted with 500 μL ACN to remove the precipitate, then mixed with 100 μL ACN containing same concentration of the reference compounds and I.S. as above and processed, and the peak area was labeled as C. The ratios of A to B and C to B were adopted for calculating recovery and matrix effect, respectively.

### Pharmacokinetic study

Rats were divided into a blank group and a FKQJF administration group (6 rats for each), and fasted for 24 h. Each rat of the administration group was given FKQJF extract suspension at a single dose of 3.2 g FKQJF/kg body weight through oral administration in parallel. Blood samples (0.25 mL) were gathered from the orbital venous plexus of each rat, with FKQJF group gathered at 14 serial timepoints (6 rats for each) as 0.083 h, 0.16 h, 0.33 h, 0.5 h, 0.75 h, 1.0 h, 1.5 h, 2 h, 3 h, 4 h, 6 h, 8 h, 12 h and 24 h after drug administration. The blood samples were centrifugated at 6000*g* for 10 min to isolate plasma and stored at − 20 °C for further analysis.

### Data analysis

Data were analyzed using the Drug and Statistics (DAS) Software version 2.0 (DAS 2.0, Mathematical Pharmacology Professional Committee of China, Shanghai, China). The non-compartmental mode was applied to obtain the following PK parameters: area under the plasma concentration–time curve from time zero to the time of last quantifiable concentration (AUC_0→t_) and area under the plasma concentration–time curve to time infinity (AUC_0→∞_), the elimination half-life (t_1/2_), mean retention time to last sampling time (MRT_0→t_) and mean retention time to infinity (MRT_0→∞_). The maximum plasma concentration (C_max_) and the time to maximum concentration (T_max_) were observed directly from the concentration–time (C–T) curves and measured data. All data are presented as mean ± SD.

## Results

### Method validation

#### Specificity

**Fig. 2:**
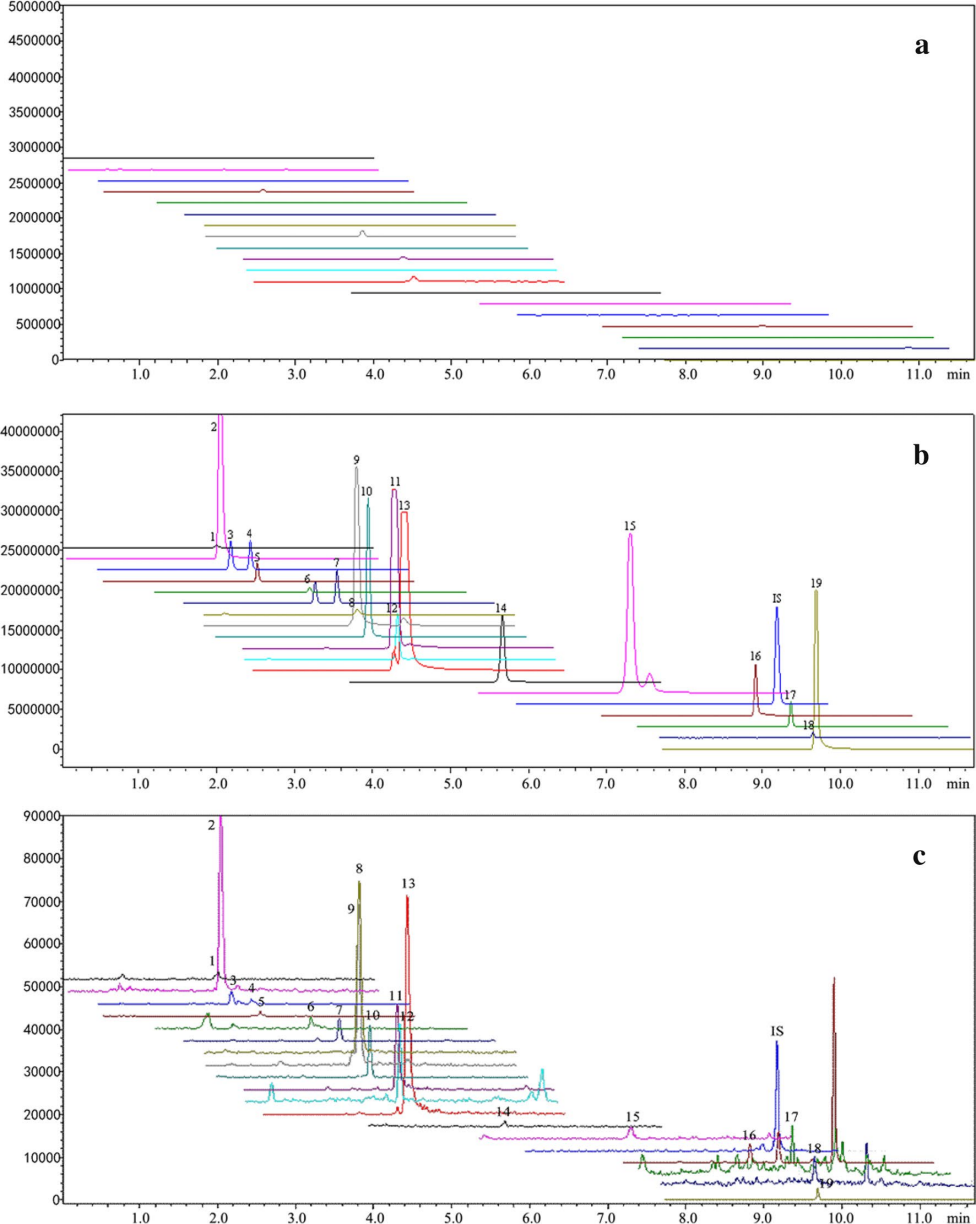
Typical MRM chromatograms of compounds **1**–**19** in FKQJF. **a** The blank plasma sample; **b** Blank plasma sample added with the reference compounds and I.S., **c** Plasma sample collected from rats after oral administration

When comparing the chromatograms of blank plasma samples, blank plasma samples added with the reference compounds containing I.S., and plasma samples after oral administration of FKQJF, we could see that all of the 19 quantifiable compounds including I.S. can be specifically detected. No endogenous substance interference was observed in rat plasma samples at the corresponding retention time of any analyte. The typical MRM chromatograms were shown in Fig. 2.

#### Linearity, LLOD and LLOQ

The established analytical method in this experiment was used to detect a series of reference compounds at different concentrations, and the linearities were obtained. The linear regression equation, correlation coefficient (*r*^2^), linear range, LLOQ and LLOD were listed in Table 2. The *r*^2^ values were between 0.9910 and 0.9994 demonstrated good linearity of regression equation of each compound. Both LLOQ and LLOD were lower than the minimum value of the linear range, indicating that the established method was suitable for quantification of FKQJF compounds absorbed into the blood.

**Table 2:** The linear regression equation, linear range, LLOQ and LLOD of 19 compounds in FKQJF

Analyte	Calibration curve	*r*^2^	Linear range (ng/mL)	LLOD (ng/mL)	LLOQ (ng/mL)
1	*y *= 0.0021*x* + 0.015	0.9993	4.88–625	0.73	2.12
**2**	*y *= 0.0906*x* + 1.9797	0.9923	4.88–625	0.85	2.08
**3**	*y *= 0.0075*x* + 0.1078	0.9941	4.88–625	0.64	1.97
**4**	*y *= 0.0053*x* + 0.062	0.9964	4.88–625	0.78	1.96
**5**	*y *= 0.0012*x* + 0.018	0.9956	4.88–625	1.25	2.58
**6**	*y *= 0.0009*x* + 0.0019	0.9991	5.81–743.75	0.68	2.45
**7**	*y *=0.0035*x *+ 0.0124	0.9910	5.13–656.25	1.24	2.32
**8**	*y *= 0.0003*x* + 0.0416	0.9940	4.88–625	1.12	2.53
**9**	*y *= 0.0816*x* + 0.6041	0.9936	1.56–200	0.23	0.76
**10**	*y *= 0.0108*x* + 0.2454	0.9926	4.88–625	0.76	2.36
**11**	*y *= 0.1335*x* + 0.6769	0.9957	2.37–303.13	0.81	1.58
**12**	*y *= 0.0007*x* + 0.015	0.9932	6.01–768.75	1.02	2.98
**13**	*y *= 0.1113*x* + 0.6035	0.9980	2.73–350	0.88	1.75
**14**	*y *= 0.0052*x* − 0.0095	0.9994	4.88–625	0.59	2.06
**15**	*y *= 0.076*x* + 0.8723	0.9954	4.88–312.5	0.92	2.43
**16**	*y *= 0.006*x* + 0.0716	0.9925	4.98–637.5	0.88	2.43
**17**	*y *= 0.0058*x* + 0.0282	0.9984	6.01–768.75	1.04	3.32
**18**	*y *= 0.0001*x* + 0.0209	0.9964	4.05–518.75	0.93	2.33
**19**	*y *= 0.0113*x* + 0.0195	0.9983	3.52–450	0.34	1.05

#### Precision and accuracy

The data of precision and accuracy were shown in Table 3. The maximum RSD values of the intra-day and inter-day precision of 19 analytes in FKQJF were 14.94% and 14.81%, respectively, indicating that the errors were within the acceptable criteria (less than 15%). Meanwhile, the intra-day accuracy ranged between 80.33 and 119.76% and the inter-day accuracy ranged between 80.03 and 119.43%. These results show that the method was feasible for this experiment.

**Table 3:** The precision and accuracy of 19 compounds in FKQJF

Analyte	Spiked (ng/mL)	Intra-day		Inter-day	
		Precision (RSD %)	Accuracy (%)	Precision (RSD %)	Accuracy (%)
**1**	500	14.94	93.93	12.08	82.52
	250	6.65	108.26	6.22	91.24
	50	13.39	113.94	8.75	85.02
**2**	500	12.48	112.91	13.95	80.94
	250	8.48	80.88	13.18	93.34
	50	6.27	96.72	5.90	90.32
**3**	500	10.61	108.40	6.47	89.09
	250	14.24	100.18	13.93	99.35
	50	8.71	82.38	4.33	97.38
**4**	500	4.84	112.84	8.43	97.25
	250	5.27	100.10	6.41	119.43
	50	9.46	87.00	8.11	88.31
**5**	600	7.81	108.38	8.47	112.68
	300	14.76	86.11	9.76	105.33
	60	11.11	110.49	14.19	103.58
**6**	600	10.81	114.84	9.43	94.40
	300	7.90	83.02	13.57	106.38
	60	10.82	108.04	8.61	90.00
**7**	500	13.68	92.90	4.31	98.35
	250	12.95	81.97	10.88	98.16
	50	5.48	96.41	5.00	101.20
**8**	500	8.68	92.52	14.81	104.02
	250	8.61	85.92	5.69	106.35
	50	10.67	117.97	8.19	99.10
**9**	150	9.92	109.50	6.29	111.14
	75	13.28	89.18	6.03	84.78
	25	10.13	87.51	5.53	80.03
**10**	500	4.92	103.61	13.94	107.44
	250	10.71	91.96	9.03	98.62
	50	13.94	102.51	5.47	81.71
**11**	200	8.87	86.93	11.32	89.26
	100	6.63	118.80	7.25	93.71
	5	6.64	107.27	4.85	96.59
**12**	600	7.59	113.80	12.96	105.22
	300	7.40	103.81	12.45	110.32
	60	7.82	118.22	11.99	109.08
**13**	200	9.81	89.49	7.92	89.26
	100	4.34	108.30	4.10	99.07
	5	8.16	92.97	13.95	108.55
**14**	500	5.45	86.37	6.65	93.67
	250	10.77	119.76	14.21	83.17
	50	4.49	83.50	9.89	119.30
**15**	600	13.10	112.68	8.06	110.97
	300	6.90	91.02	10.91	88.92
	60	13.93	103.81	9.02	92.39
**16**	500	5.70	85.56	13.38	87.62
	250	11.61	89.73	14.78	100.02
	50	9.72	118.83	10.08	96.61
**17**	600	8.29	109.97	7.57	95.28
	300	4.62	102.48	4.25	93.29
	60	14.85	90.07	4.57	99.00
**18**	400	4.68	100.57	12.66	109.09
	200	10.44	115.16	10.62	87.68
	20	12.41	116.16	13.41	87.29
**19**	300	4.83	115.96	13.09	91.69
	150	7.15	106.77	9.09	115.94
	10	6.41	80.33	11.85	89.01

#### Stability

The stability RSD values of plasma samples and reference compounds containing I.S. were all less than 15% after being placed at room temperature for 8 h, freeze-thawed 3 times, or stored at low temperature of − 20 °C for 20 days. Data shown in Table 4 presented good stability in the process of sample preparation and storation.

**Table 4:** The stability of 19 compounds in FKQJF

Analyte	Spiked (ng/mL)	Ordinary stability		Freeze–thaw stability		Long-term stability	
		Mean (%)	RSD (%)	Mean (%)	RSD (%)	Mean (%)	RSD (%)
**1**	500	117.94	11.54	106.56	7.03	103.76	12.98
	250	104.48	8.53	99.57	5.60	89.00	6.73
	50	80.92	10.50	112.34	7.15	86.86	11.68
**2**	500	95.34	14.84	86.48	6.00	112.17	7.31
	250	86.35	8.62	99.07	5.02	83.50	14.58
	50	106.33	9.23	98.92	4.81	87.08	8.91
**3**	500	109.98	11.54	90.89	7.53	93.19	6.90
	250	84.19	8.09	92.61	13.98	86.77	9.78
	50	93.52	12.39	84.84	6.45	81.68	14.16
**4**	500	84.20	5.31	102.81	7.52	104.85	7.34
	250	116.00	5.04	107.25	7.93	81.20	9.27
	50	108.94	8.11	91.90	5.35	118.34	5.70
**5**	600	94.88	10.89	113.31	8.05	82.61	8.14
	300	103.80	5.21	80.22	4.40	97.44	9.91
	60	81.32	11.81	109.12	7.42	113.28	4.21
**6**	600	91.44	14.45	88.33	12.69	102.00	14.51
	300	109.93	12.98	102.20	12.84	91.27	7.41
	60	88.22	8.15	115.63	7.36	83.07	6.13
**7**	500	109.98	11.54	90.89	7.53	97.81	11.96
	250	84.19	8.09	92.61	13.98	104.72	5.20
	50	93.52	12.39	84.84	6.45	118.09	13.14
**8**	500	103.60	11.87	88.21	10.59	87.97	7.21
	250	108.42	9.44	102.50	8.24	114.13	11.46
	50	95.42	14.51	80.11	11.62	109.80	5.76
**9**	150	92.51	13.34	113.07	8.98	98.06	13.75
	75	93.01	6.11	93.19	6.90	91.97	4.96
	25	104.79	12.65	86.77	9.78	80.63	9.89
**10**	500	103.90	7.69	81.68	14.16	107.95	7.12
	250	82.57	11.25	102.95	7.36	81.88	12.37
	50	95.59	9.28	118.81	13.10	95.03	9.95
**11**	200	112.88	4.17	86.16	10.51	89.65	10.46
	100	81.64	9.85	98.74	14.77	101.38	11.50
	5	92.36	14.37	94.51	12.37	87.02	7.26
**12**	600	85.93	4.56	93.19	4.61	96.64	14.09
	300	107.50	8.26	86.83	6.24	94.37	7.35
	60	115.56	13.37	84.52	8.98	115.89	10.17
**13**	200	113.97	5.23	104.87	6.41	114.48	6.54
	100	103.56	9.41	110.44	4.41	81.78	7.88
	5	84.57	12.21	97.72	11.87	102.75	5.51
**14**	500	101.11	11.28	84.71	9.66	99.51	14.31
	250	92.68	8.37	116.86	10.11	103.10	5.36
	50	80.39	14.63	88.34	7.21	91.66	12.00
**15**	600	82.90	5.58	118.41	13.88	88.35	11.17
	300	119.43	8.54	105.94	5.17	98.43	11.78
	60	89.92	7.89	114.11	5.74	100.55	9.41
**16**	500	88.55	10.19	95.34	14.39	95.48	14.48
	250	92.98	9.71	108.03	7.63	89.82	11.06
	50	83.35	10.11	105.76	13.36	105.80	14.99
**17**	600	89.06	13.56	115.83	10.90	117.51	8.16
	300	117.99	8.45	86.68	7.22	90.66	13.65
	60	81.80	13.15	82.73	12.65	87.43	14.67
**18**	400	106.31	5.16	104.63	14.09	118.76	7.32
	200	86.65	11.80	112.10	12.30	86.82	11.71
	20	107.46	10.78	88.16	4.78	114.56	10.35
**19**	300	96.04	14.20	94.97	5.56	85.96	7.14
	150	80.66	4.23	111.86	11.51	84.29	11.25
	10	96.33	12.53	103.66	13.37	116.14	5.09

#### Recovery and matrix effect

As shown in Table 5, the extraction recovery and matrix effect of the analytes with different concentrations ranged from 80.71 to 119.90% and from 80.43 to 119.79%, respectively, indicating an acceptable recovery and an analysis method without obvious matrix effect.

**Table 5:** The recovery and matrix effect of 19 compounds in FKQJF

Analyte	Spiked (ng/mL)	Recovery		Matrix effect	
		Mean	RSD %	Mean	RSD %
**1**	500	91.34	8.16	83.40	4.46
	250	117.65	10.34	86.26	13.09
	50	95.57	9.65	95.83	12.51
**2**	500	82.25	6.95	95.64	14.43
	250	88.18	5.16	118.82	9.91
	50	95.72	13.70	83.95	13.26
**3**	500	112.88	4.17	86.16	10.51
	250	81.64	9.85	98.74	14.77
	50	92.36	14.37	94.51	12.37
**4**	500	96.00	10.04	109.41	10.74
	250	112.34	5.75	96.07	7.33
	50	93.48	12.31	81.89	7.77
**5**	600	116.92	7.90	99.37	11.80
	300	93.25	7.38	81.76	7.84
	60	113.16	11.01	90.40	14.03
**6**	600	116.07	14.09	95.91	5.33
	300	90.96	9.27	101.58	7.86
	60	113.50	10.86	83.42	4.92
**7**	500	94.80	10.92	83.83	4.18
	250	84.24	13.75	80.63	11.34
	50	86.90	14.70	105.95	14.40
**8**	500	114.26	10.58	106.24	11.57
	250	97.53	7.22	91.89	8.13
	50	82.58	8.09	100.86	4.65
**9**	150	88.02	5.08	115.13	5.53
	75	84.66	4.90	114.37	9.09
	25	97.35	5.71	96.57	11.85
**10**	500	114.19	12.24	112.83	4.96
	250	119.00	13.92	116.16	13.93
	50	93.86	11.51	88.16	4.67
**11**	200	103.16	5.57	88.83	6.67
	100	100.81	5.27	102.26	6.90
	10	90.40	12.70	80.62	11.88
**12**	600	113.68	8.76	114.11	8.79
	300	94.96	14.89	85.75	7.80
	60	111.70	4.41	119.18	11.76
**13**	200	94.08	14.89	119.79	12.86
	100	114.75	9.32	80.43	4.68
	10	85.57	11.82	85.26	6.47
**14**	500	119.18	6.16	83.89	6.95
	250	112.28	7.14	84.01	9.14
	50	119.90	10.03	114.12	7.35
**15**	600	82.03	6.08	91.20	8.50
	300	80.71	5.05	106.55	11.58
	60	82.33	7.78	96.12	12.48
**16**	500	82.87	9.34	113.66	4.04
	250	96.22	9.00	100.13	14.14
	50	101.27	11.48	88.82	14.28
**17**	600	91.18	11.92	89.61	9.44
	300	90.15	10.63	99.43	10.45
	60	101.00	10.52	86.38	9.42
**18**	400	110.60	6.17	83.36	9.40
	200	96.94	9.84	98.95	9.81
	20	99.16	9.57	96.26	12.07
**19**	300	88.37	8.76	83.94	8.74
	150	95.79	5.85	86.30	11.95
	10	103.73	7.60	102.13	4.23

### Pharmacokinetic study

**Fig. 3:**
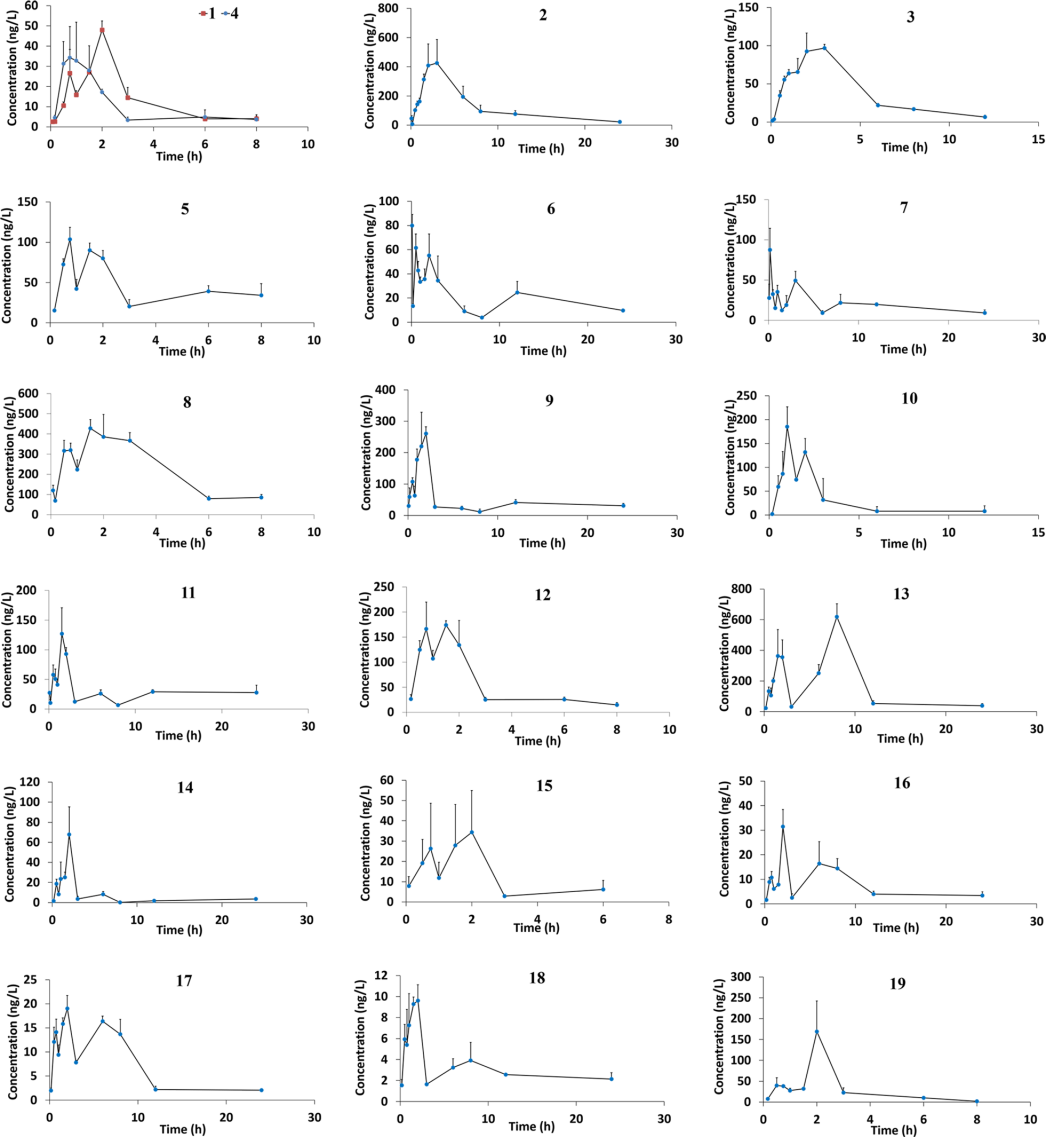
The plasma concentration–time curves of 19 FKQJF compounds

Plasma samples were prepared according to the method in section of “Pretreatment of plasma samples” and analyzed by using the established method. The blood concentration–time curves of the compounds were shown in Fig. 3, and the pharmacokinetic (PK) parameters were shown in Table 6.

**Table 6:** The main pharmacokinetic parameters of 19 FKQJF compounds in plasma of rats

Analyte	AUC_0→t_ (ng h/mL)	AUC_0→∞_ (ng h/mL)	MRT_0→t_ (h)	MRT_0→∞_ (h)	t_1/2_ (h)	T_max_ (h)	C_max_ (ng/mL)
**1**	108.74 ± 11.40	115.09 ± 15.76	2.60 ± 0.12	3.37 ± 0.26	1.79 ± 0.44	2.00 ± 0.00	47.99 ± 4.41
**2**	2967.61 ± 169.49	3110.48 ± 38.66	6.51 ± 0.66	8.09 ± 1.32	5.27 ± 2.94	2.17 ± 0.76	477.02 ± 33.31
**3**	463.00 ± 29.50	489.12 ± 37.60	3.67 ± 0.14	4.32 ± 0.47	2.66 ± 0.69	2.33 ± 0.58	101.53 ± 18.42
**4**	80.47 ± 9.64	92.61 ± 21.43	2.38 ± 0.24	3.25 ± 0.41	2.20 ± 1.24	1.00 ± 0.50	46.84 ± 6.78
**5**	344.22 ± 43.08	504.72 ± 108.63	3.51 ± 0.24	7.55 ± 1.93	3.89 ± 0.85	1.00 ± 0.43	103.90 ± 14.93
**6**	466.39 ± 51.94	1012.04 ± 558.43	9.19 ± 1.00	13.95 ± 9.49	10.49 ± 6.63	0.08 ± 0.00	79.85 ± 9.43
**7**	468.14 ± 48.36	550.30 ± 70.65	9.04 ± 0.68	14.25 ± 3.28	7.68 ± 3.01	0.17 ± 0.00	87.65 ± 26.65
**8**	1800.70 ± 154.66	2142.54 ± 167.73	2.88 ± 0.15	4.31 ± 0.67	2.70 ± 0.54	1.67 ± 0.29	432.72 ± 53.10
**9**	1090.97 ± 109.82	2548.08 ± 277.22	8.96 ± 0.40	10.52 ± 4.72	9.65 ± 4.91	1.83 ± 0.29	269.68 ± 24.01
**10**	369.68 ± 155.83	374.65 ± 153.08	2.54 ± 1.12	3.13 ± 1.66	1.64 ± 0.52	1.33 ± 0.58	192.33 ± 31.98
**11**	691.13 ± 93.26	1638.89 ± 155.01	11.02 ± 0.99	14.16 ± 8.89	9.16 ± 2.09	1.67 ± 0.29	133.90 ± 31.78
**12**	441.56 ± 59.51	480.47 ± 71.86	2.45 ± 0.19	3.10 ± 0.33	1.79 ± 0.20	1.00 ± 0.43	186.20 ± 19.02
**13**	3803.21 ± 170.75	5627.52 ± 2430.69	8.04 ± 0.88	39.04 ± 46.48	38.76 ± 53.16	8.00 ± 0.00	619.12 ± 84.57
**14**	143.85 ± 16.54	205.76 ± 58.66	6.67 ± 1.42	17.02 ± 8.17	12.11 ± 9.32	2.00 ± 0.00	67.74 ± 27.55
**15**	74.29 ± 17.48	81.11 ± 19.35	2.21 ± 0.48	3.05 ± 1.01	1.20 ± 0.74	1.42 ± 0.63	47.74 ± 5.92
**16**	177.10 ± 17.10	250.82 ± 97.56	8.68 ± 1.17	20.144 ± 5.01	12.64 ± 12.72	2.00 ± 0.00	31.45 ± 6.94
**17**	160.80 ± 12.69	178.64 ± 14.30	7.30 ± 0.38	9.82 ± 0.45	5.98 ± 0.40	3.33 ± 2.31	19.20 ± 2.48
**18**	74.65 ± 8.31	193.63 ± 27.92	9.85 ± 0.85	48.18 ± 39.82	33.59 ± 27.13	1.83 ± 0.29	10.19 ± 0.58
**19**	248.66 ± 65.48	251.96 ± 62.45	2.44 ± 0.12	2.55 ± 0.21	1.10 ± 0.47	2.00 ± 0.00	169.08 ± 73.48

The results in Fig. 3 show that all the 19 compounds could be rapidly absorbed by the gastrointestinal tract after the extract of FKQJF were orally administered to rats at a single dose of 3.2 g/kg body weight, the concentration of which in plasma could be determined about 10 min later, and the plasma drug concentrations of most compounds reached the peak values at about 1–2 h. Two compounds, berberine (**13**) and andrograpanin (**17**), displayed peak times longer than 3 h (the T_max_ were 8.00 ± 0.00 and 3.33 ± 2.31, respectively), indicating the gastrointestinal absorption of these two compounds was lower than that of other ones. The plasma concentrations of other compounds, 5-hydroxypicolinic acid methyl ester (**2**), acortatarin A (**3**), 9-epi-acortatarin A (**4**), jatrorrhizine (**9**), palmatine (**11**), naringenin (**14**) and 7-*O*-methylwogonin (**19**) decreased rapidly with the time prolongation after reaching peak values but could still be determined in plasma after a period. This result could be inferred that the absorption of this kind of compounds has the characteristics of fast absorption, fast elimination and long residence time. C_max_ and AUC of 5-hydroxypicolinic acid methyl ester (2), salicylic acid (8), jatrorrhizine (9), palmatine (11) and berberine (13) were higher than the other compounds. However, in our previous study the oral absorption (C_max_) of alkaloids (jatrorrhizine, palmatine and berberine) was relatively low, possibly due to complex drug interaction in a Chinese medicine formula [18]. By comparing the pharmacokinetic characteristics of acortatarin A (3) and 9-epi-acortatarin A (4), although the half-life of both was close (t_1/2_ was 2.66 and 2.20 h respectively), the AUC and C_max_ of acortatarin A were much higher than 9-epi-acortatarin A which indicated the bioavailability of 9-epi-acortatarin A was lower than acortatarin A due to its relatively small exposure in the body. It can be seen that the structural characteristics of compounds itself influenced its absorption in vivo to a certain extent [19].

In addition, the concentration–time curves of 12 compounds including 3,4,5-trimethoxyphenol-1-*O*-β-d-glucopyranoside (**1**), neoandrographolide (**5**), *trans*-ferulic acid (**6**), genistin (**7**), salicylic acid (**8**), ononin (**10**), andrographolide (**12**), berberine (**13**), 14-deoxy-11,12-didehydroandrographolide (**15**), panicolin (**16**), andrograpanin (**17**), and *Z*-ligustilide (**18**) in this experiment showed double-peaks, which means that the plasma drug concentration reached the peak for the first time, and decreased with the time going but rose again to form the second peak. The bimodal phenomenon of these compounds may be related to the enterohepatic circulation [20–22], distribution [23], variable gastric emptying and double-site absorption [24, 25] during the absorption process of these compounds. As shown in Fig. 3 and Table 6, owing to the very similar structures, except for andrograpanin the other three diterpenes (neoandrographolide, andrographolide and 14-deoxy-11,12-didehydroandrographolide) have similar pharmacokinetic parameters and concentration–time curves in vivo, being absorbed and eliminated with the similar rate. The first peak of the three diterpenes arises at 0–1 h, and the second peak appears 1.5–3 h post dose. As showed in Fig. 1, neoandrographolide is the derivative of andrograpanin, in which the hydroxyl group is substituted by glucose, but their pharmacokinetic parameters are remarkably different, with T_max_ at 1.00 h, neoandrographolide was absorbed more rapidly than andrograpanin (T_max_ at 3.33 h). Notably, distinct double-peaks of the four diterpenes were observed in plasma concentration–time curves of all these timosaponins, which was in conformity with the previous study [26]. However, the factors that may contribute to double peaks are not clear and the specific reasons need to be further studied. Double plasma concentration peaks were also observed in the plasma concentration curves profiles of berberine, which was consistent with results from previous studies [27]. Distribution re-absorption and enterohepatic circulation may contribute to double peaks phenomenon. It was reported that while the berberine was absorbed, it was distributed rapidly with higher concentration in tissues than that of the plasma, which is possible for the drug to transfer from tissues to plasma and causes another peak in plasma [28]. Another possible cause of bimodal phenomena is that berberine inhibited gastrointestinal peristalsis which influenced drug absorption [29]. Moreover, one glucoside (3,4,5-trimethoxyphenol-1-*O*-β-d-glucopyranoside) [18], a flavone (genistin) [30] and a phthalide (Z-ligustilide) [31] presented a double-peak absorption phase, which were reported. It is well known that the absorption of compounds in TCM is a very complex process that manifests itself through potential interaction with a host of physicochemical and physiological variables. Therefore, further detailed absorption studies are needed to elucidate the mechanism of the double-peak phenomenon in pharmacokinetics.

## Discussion

In our previous study, we systematically studied the chemical constituents of FKQJF as well as the single herbs, *Mahoniae Caulis* and *Spatholobi Caulis* [14, 15]. We also established a high-performance liquid chromatography method for fingerprint and simultaneous quantification of eight major compounds in Fukeqianjin capsule [32]. Moreover, our group has already studied the PK behaviors of *Mahoniae Caulis* and *Spatholobi Caulis*, which is a significant foundation to investigate the PK study of the formula more effectively [18, 33]. On the basis of compound chemical components research of FKQJF and its vital components, we studied the major effective compounds observed in blood through the pharmacokinetics research, which are the mostly possible material basis of FKQJF. These studies will provide helpful information for clarifying bioactive constituents and action mechanism of this TCM prescription.

## Conclusions

With the rapid development of analytical techniques, LC–MS is increasingly used in the analysis of complex TCM systems [33, 34]. UFLC–MS/MS technology combines the advantages of the rapid separation of liquid chromatography, accurate molecular weight measurement of mass spectrometry along with fragmented ion information to pinpoint and analyze the ng/mL range of compounds accurately. It provided an accurate and reliable analytical method for the determination of various compounds in TCM and their pharmacokinetics of absorption, distribution, metabolism and excretion in vivo [35–37]. In this study, a rapid, efficient and sensitive method for detecting the absorption behavior of the compounds in Fukeqianjin formula was established based on the characteristics of its components. The PK behaviors of 19 compounds absorbed into the blood, including 4 diterpenoids, 6 alkaloids, 5 flavones, 1 lactone and 3 organic acids, were determined using this method, and the key kinetic parameters, such as T_max_, half-life, AUC and MRT were obtained. The result of this experiment could help further research on promotion of the pharmacological activity and dosage form design of FKQJF, as well as potential clinical application of its active compounds, and provides a key link between the complex chemical system of FKQJF and its in vivo activities.

## Data Availability

All data included in this article are available from the corresponding author upon request.

## Abbreviations

UFLC–MS/MS: Ultra-fast liquid chromatography coupled with triple quadrupole tandem mass spectrometry
FKQJF: Fukeqianjin formula
TCM: Traditional Chinese medicine
I.S.: Internal standard
MRM: Multiple reaction-monitoring
LLOD: Lower limit of detection
LLOQ: Lower limit of quantitation
RSD: Relative standard deviation
QC: Quality control
PK: Pharmacokinetic
MeOH: Methanol
CAN: Acetonitrile
SD: Sprague–Dawley
ESI: Electrospray ionization source
